# Identity-Based Efficient Secure Data Communication Protocol for Hierarchical Sensor Groups in Smart Grid

**DOI:** 10.3390/s25164955

**Published:** 2025-08-10

**Authors:** Yun Feng, Yi Sun, Yongfeng Cao, Bin Xu, Yong Li

**Affiliations:** 1 China Electric Power Research Institute, Beijing 100192, China; fengyun67067@163.com (Y.F.); caoyf175@126.com (Y.C.); xubin36277888@163.com (B.X.); 2 School of Electrical and Electronic Engineering, North China Electric Power University, Beijing 102206, China; 3 State Grid Shandong Electric Power Institute, Jinan 250000, China; 13853105727@163.com

**Keywords:** smart grid, identity-based encryption, lightweight authentication, dynamic key management, hierarchical sensors

## Abstract

With the rapid evolution of smart grids, secure and efficient data communication among hierarchical sensor devices has become critical to ensure privacy and system integrity. However, existing protocols often fail to balance security strength and resource constraints of terminal sensors. In this paper, we propose a novel identity-based secure data communication protocol tailored for hierarchical sensor groups in smart grid environments. The protocol integrates symmetric and asymmetric encryption to enable secure and efficient data sharing. To reduce computational overhead, a Bloom filter is employed for lightweight identity encoding, and a cloud-assisted pre-authentication mechanism is introduced to enhance access efficiency. Furthermore, we design a dynamic group key update scheme with minimal operations to maintain forward and backward security in evolving sensor networks. Security analysis proves that the protocol is resistant to replay and impersonation attacks, while experimental results demonstrate significant improvements in computational and communication efficiency compared to state-of-the-art methods—achieving reductions of 73.94% in authentication computation cost, 37.77% in encryption, and 55.75% in decryption, along with a 79.98% decrease in communication overhead during authentication.

## 1. Introduction

With the wide application of machine learning and network technology, the power system has been rapidly developed, forming the idea of the smart grid consisting of multilayer smart sensors. Smart grids leverage and aggregate information collected by sensors to assist in making power management decisions [1,2]. As a new generation of power systems, the smart grid provides users with stable and reliable power services and achieves efficient operation and intelligent management [3,4]. However, since electric power information is relevant to user privacy, data communication among various sensors and the power service center leads to significant security concerns [5].

To achieve efficient communication while guaranteeing data privacy in smart grid systems, existing secure data communication protocols [6,7] combine asymmetric and symmetric encryption algorithms. With the high efficiency of symmetric encryption methods [8,9], sensitive electric data is masked by a predetermined symmetric secret key, which is only accessed by authorized groups. Due to the security of asymmetric encryption methods [10], a pair of asymmetric secret keys is utilized to share the symmetric secret key in a smart grid system.

Recently, identity-based encryption is one of the most effective asymmetric encryption methods for authentication among dynamic hierarchical sensor groups [11]. Specifically, the identity-based encryption method utilizes identity information to guarantee that only authorized users can obtain valuable information from ciphertext. Gupta et al. [12] propose an efficient identity-based protocol for authentication in transport systems. Zhao et al. [13] further introduce an identity-based broadcast signcryption scheme in the Internet of Vehicles. Shen et al. [14] focus on security enhancement and present an identity-based higncryption protocol. Particularly, for hierarchical architecture scenario applications, Pavithran et al. [15] propose a blockchain-aided protocol to utilize hierarchical identity-based encryption in the Internet of Things systems and Badar et al. [16] propose an identity-based authentication protocol using the physical unclonable function, particularly for smart grid scenarios.

However, these data encryption methods involve complex computations, such as pairing and modular inversion operations. In practice, smart sensors deployed on the terminal side have limited computing resources, while data communication and collaborative analysis require real-time feedback. To match the requirements of real-time grid data processing, developing a secure data communication protocol with efficiency improvement has become a hot topic.

Additionally, the dynamic sensor group leads to a huge cost for secret group key updates [17], particularly in smart grid scenarios. On the one hand, the dense update frequency of the sensor group has a significant impact on the performance of the smart grid system. On the other hand, the change of group members causes forward and backward security concerns for the secret group key.

Based on the analysis above, this paper proposes an identity-based efficient secure data communication protocol for hierarchical sensor groups in smart grid systems. The main contributions of this paper can be summarized in three aspects:

1. We propose a novel secure data communication protocol for sensor groups in the smart grid system, leveraging the symmetric encryption method to transmit obscured data and the identity-based encryption method to share group secret keys. Identities of authorized users are encoded by Bloom filter, and a cloud-aided pre-verification procedure is introduced. Efficient authentication is achieved by searching the pre-calculated authentication array table in the cloud server.
2. A dynamic update mechanism of the group secret key is designed corresponding with the proposed protocol for lower resource costs in smart grid scenarios. When the sensor group is changed, the proposed mechanism utilizes lightweight operations to implement dynamic updates of the group secret key, which guarantees forward and backward security for the smart grid system.
3. Theoretical analysis demonstrates that our protocol achieves forward and backward security of a dynamic sensor group and has the capability to resist the replay attack and impersonation attack. Experimental evaluation indicates that our protocol performs better than the state-of-the-art protocols. Specifically, for computation cost, the proposed protocol is 73.94% superior to others on average in the authentication process, 37.77% in the encryption process, and 55.75% in the decryption process. For communication cost, the proposed protocol is 79.98% superior to others on average in the authentication process.

The remainder of this paper is organized as follows: The subsequent section presents current research work relevant to this study. Section 3 introduces the system model. The definition related to this study is detailed in Section 4. Section 5 introduces the specific content of this protocol. Section 6 is the security analysis of this protocol, and Section 7 introduces performance analysis. The concluding section encapsulates the research presented in this paper.

## 2. Related Works

### 2.1. Broadcast Encryption Algorithm

Broadcast encryption was first proposed in 1993 [18]. Recently, Boneh et al. [19] proposed a broadcast encryption scheme using bilinear mapping. The private key and ciphertext of the scheme reached the constant level, which supports the anti-collusion attack and proves that the broadcaster could no longer be a trusted authority but any legitimate user. However, the number of users for this solution has been set at the beginning of the solution, and subsequent users cannot be added. Lewko et al. [20] proposed a broadcast encryption scheme that supports the user’s cancellation and non-monotonic mechanism, making the application of broadcast encryption more flexible. However, although the above scheme and subsequent schemes [21,22] realize the advantages of multiple users sharing the same message, they cannot achieve good access control. Kumar [23] et al. proposed a broadcast encryption technology based on threshold and wildcard. This technique uses hidden access policies to provide security for sensitive data broadcast over insecure channels. In addition, for any number of attributes, the technology realizes the fixed-length ciphertext, which greatly reduces the communication overhead and computational complexity. However, the proposed scheme does not consider the direct withdrawal of users and the dynamic addition of users in the system.

### 2.2. Authentication Protocol

Wazid et al. [24] proposed a three-factor authentication protocol for remote users in a smart grid environment based on renewable energy. The protocol uses one-way hash functions, bitwise OR operations, and ECC operations to achieve lightweight encryption. The protocol supports the dynamic addition of smart sensors, flexibility of password and biometric updates, user anonymity, and non-traceability. Mahmood et al. [25] proposed a lightweight authentication protocol based on ECC, which used ProVerif, an automatic verification tool, to analyze its own security, and adopted Burrows–Abadi–Needham (BAN) logic to prove the completeness and completeness of the protocol. The downside is that the protocol does not support the anonymity of smart sensors. Kumar et al. [26] proposed a lightweight authentication and key agreement protocol that achieves anonymity, integrity, and security based on ECC, symmetric encryption, hash functions, and message authentication codes. Wang et al. [27] proposed a mutual authentication protocol based on edge computing in a smart grid system, which supports efficient conditional anonymity and key management based on blockchain technology. The proposed protocol ensures mutual authentication and anti-replay attacks and supports efficient key renewal and revocation to achieve conditional anonymity with lower computing and communication costs.

### 2.3. Key Updating Protocol

Group multicast effectively improves the efficiency of group communication. How to generate and update multicast keys efficiently has important practicality and wide application prospects in the smart grid. At present, a large number of group key generation and updating protocols have been proposed, which can be divided into three categories: key updating protocol based on binary key tree, key updating protocol based on multi-fork key tree, and key updating protocol based on polynomial. Lin et al. [28] proposed an M-fork tree key management and digital signature protocol based on elliptic curves. At the time of transmission, the protocol provides several flexible and scalable schemes to manage security issues that dynamically adapt the framework to the rapidly changing IoV topology, speeding up the time to synchronize system key reconstruction and reducing the number of stages to resynchronize system keys. Tan et al. [29] proposed a dynamic key management scheme based on attribute-based encryption. In the vehicular ad hoc network, identity authentication plays an important role in privacy protection. This protocol guarantees non-repudiation and authenticity properties while achieving efficient vehicular communications.

## 3. System Model

**Figure 1:**
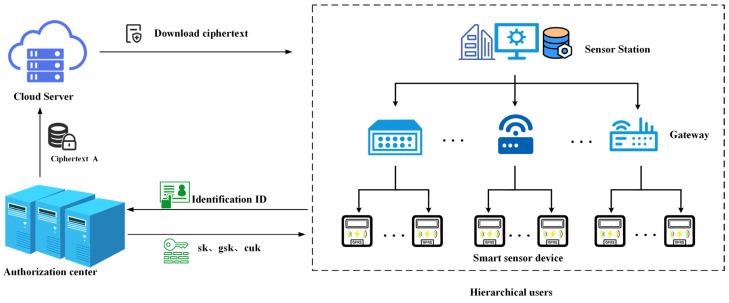
System model of the proposed secure data communication protocol.

As shown in Figure 1, the system model consists of three entities: cloud server, authorization center, and smart sensor devices.

**Cloud server:** As one of the components of the power service center, the cloud server has sufficient computing resources and storage capacity. It is responsible for storing ciphertext and performing pre-authentication operations for device access requests. Additionally, the cloud server takes responsibility for removing inactive devices that have not established a connection beyond a predefined threshold, thereby effectively eliminating broken-down devices.

**Authorization center:** The authorization center is a trusted entity that is responsible for generating system parameters, public and private key pairs, user keys, and session keys based on the identity of the smart device. The authorization center sends them to the smart device. In addition, the authorization center is responsible for the plaintext encryption to generate the ciphertext and send it to the cloud server. Additionally, the authorization center conducts regular checks for the cloud server, implementing maintenance procedures or server replacement when an abnormal status is detected.

**Smart sensor device:** The smart sensor devices consist of a master station system, gateway devices, and smart sensors. The smart sensor device has limited computing power and storage capabilities, and every device has a public–private key pair, a user key, and a session key. The smart sensor devices use the session key to communicate with the power service center and decrypt the ciphertext using their private keys.

## 4. Definition

### 4.1. Bloom Filter

A Bloom filter is a random data structure with high special efficiency. The set $S=\{x_{1},x_{2},\ldots ,x_{n}\}$ is encoded in an array of W bits and the Bloom filter determines whether an element x belongs to the set S. The steps to build the (w, m, k, H) Bloom filter BFS for set S are as follows. First, the set of hash functions $H=\{h_{0},h_{1},\ldots ,h_{k-1}\}$ is selected, where the hash functions $h_{0},h_{1},\ldots ,h_{k-1}$ are independent of each other and have the range [0,w−1]. All bits of BFS are then set to 0 initially. Finally, for all x∈S and 0≤i≤k−1, let $BF\left(h_{i}\left(x\right)\right)=1$. However, the Bloom filter may misjudge when determining whether the element belongs to the set S by mistaking x∉S for x∈S. When it is necessary to determine whether the element y belongs to the set S, simply compute $h_{i}(y)(0\leq y\leq k-1)$ and check $BF(h_{i}(y))$ whether all values are 1. If the result is not all 1, y∉S, otherwise y∈S. The maximum error rate is

$$\varepsilon =p^{k}(1+O(\frac{k}{p}\sqrt{\frac{\ln w-kln\;p}{w}})),$$

where $p=1-{\left(1-\frac{1}{w}\right)}^{km}$ and ε is a negligible function of k.

### 4.2. Identity-Based Public Key Encryption Algorithm

The identity-based cryptosystem makes use of the bilinear property of elliptic curves, and bilinear pairing is established by the relation between cyclic subgroups of elliptic curves and multiplicative cyclic subgroups of extended domains. When the difficulty of the extended domain discrete logarithm problem is similar to that of the elliptic curve discrete logarithm problem, an efficient and secure identity-based cryptosystem can be constructed. The identity-based data encryption algorithm is defined as follows:

P1 is set as the generator of the elliptic curve addition cyclic group G1, and P2 is the generator of the elliptic curve addition cyclic group G2. H (·) stands for hash function; Enc (·) and Dec (·), respectively, correspond to the operation modes of encryption and decryption. KDF (·) is a function involved in the key derivation process; MAC (·) is the authentication message code that carries the key in the authentication process; e (·) is a bilinear pair.

The cryptographic function $H_{1}(Z,n)$ takes the bit string Z and an integer n and outputs an integer $h_{1}\in [1,n-1]$. The cryptographic function $H_{2}(Z,n)$ takes the bit string Z and an integer n and outputs an integer $h_{2}\in [1,n-1]$. The key generation center randomly selects ke∈[1,N−1] as mater secret key, and computes $P_{pub}=[ke]P_{1}$ in $G_{1}$ as mater public key. The encryption master key pair is  (ke, Ppub). The key generation center is kept secret at *ke* and publicly available at *Ppub*. The key generation center chooses and exposes the one-byte private key to generate the function identifier hid. The identity of the user A is ${ID}_{A}$, and generates A’s private key $d_{A}$. The key generation center first computes $t_{1}=H_{1}({ID}_{A}|\left|hid,N\right)+ke$ in the bounded domain $F_{N}$. If $t_{1}=0$, the system needs to re-generate the master private key, compute and expose the master public key, and update the existing user’s private key; otherwise, calculate $t_{2}=ke\cdot t_{1}^{-1}$, then calculate $d_{A}=\left[t_{2}\right]P_{1}=[S/(H_{1}({ID}_{A}|\left|hid\right)+s)]P_{1}$.

Assume that user A encrypts plaintext and sends it to user B, A computes element $Q_{B}=[H_{1}\left({ID}_{B}|\left|hid,N\right)\right]P_{1}+P_{pub}$ of the group $G_{1}$, and then randomly selects r∈[1,N−1], and computes $C_{1}=\left[r\right]Q_{B},$ $g=e\left(P_{pub},P_{2}\right),w=g^{r}$. A calculates $K=KDF(C_{1}|\left|w\right||{ID}_{B})$. According to the classification of encryption, there are two ways to encrypt plaintext to generate $C_{2}$: stream cipher $C_{2}=M+K$ and block cipher $C_{2}=Enc(M,K)$. A computes $C_{3}=MAC(K,C_{2})$, and finally obtains the ciphertext $C=C_{1}\left|\left|C_{2}\right|\right|C_{3}.$

After receiving the ciphertext, user B calculates $w^{\prime }=e(C_{1},{ID}_{B})$, $K^{\prime }=KDF(C_{1}|\left|w^{\prime }\right||{ID}_{B})$. According to the classification of encryption, there are two ways to decrypt ciphertext to generate $M^{\prime }$: stream cipher $M^{\prime }=C_{2}+K_{1}$ and block cipher $M^{\prime }={Dec(C}_{2}+K_{1}$). Finally, B computes $u=MAC(K_{2}^{\prime },C_{2})$. If $u=C_{3}$, output $M^{\prime }$, otherwise an error is reported.

## 5. Protocol

This protocol mainly includes six steps, which are system initialization, key generation, data encryption, user authentication, data decryption, and dynamic updating of terminal devices. The following is the specific interaction process.

### 5.1. System Initialization

Given security parameter λ, the authorization center (CA) selects a particular elliptic curve $E_{q}(a,b)$ and a point P of large prime order on an elliptic curve on a finite field, selects $G_{1}{,G}_{2}$ as addition cyclic groups of prime N, and $G_{T}$ selects a multiplicative cyclic group of primes N. Select Bloom filter’s bit array size m and k hash functions which map every identity in the user’s set to {1,2,…,m}. Generally, k hash functions are used to calculate the elements in the authorized user set, and the obtained results are modulo m, and the Bloom filter’s pre-authentication array table A is obtained and stored in the server.

### 5.2. Key Generation

CA selects a number x∈[1,N−1] at random as system master key msk, and the corresponding system master public key mpk=x·P is disclosed. CA selects and exposes the private key generating function identifier hid, which is represented by a single byte. AC computes $t_{i}^{j}=H_{1}(ID_{i}^{j}||hid,N)+x$ over a finite field. If $t_{i}^{j}\neq 0$, calculate the user’s private key ${sk}_{i}^{j}=x\cdot ({t_{i}^{j})}^{-1}$, and the corresponding user’s public key is ${pk}_{i}^{j}=x\cdot ({t_{i}^{j})}^{-1}\cdot P$. If $t_{i}^{j}=0$, the system master key and public key need to be recalculated. CA randomly selects $R_{i}^{j}$, timestamp T, the system master public key mpk, the system private key msk, and user identification ${ID}_{i}^{j}$ to generate the user key $K_{i}^{j}=H(msk||ID_{i}^{j}||R_{i}^{j}||T)$. In addition, KDC generates a random number $rand_{i}^{j}$ for every smart sensor device to generate session key $uk_{i}^{j}=H_{1}(K_{i}^{j}||rand_{i}^{j})$.

**Broadcast key generation:** For every smart sensor or gateway device, CA generates the broadcast key gsk=KGen(msk,GID) through the master public key mpk, the master private key msk and $GID=H_{1}(ID_{1}^{1}||\ldots ||ID_{m}^{n})$ of all devices, where n represents the last layer, and m represents the last smart sensor device at the last layer. CA selects grand at random to generate the broadcast session key $guk=H_{1}(gsk||grand)$.

**Multicast key generation:** When the power service center wants to multicast with the subset of users, the user key and the hash value of user identities are calculated as the multicast key:

$$csk=H_{1}(H_{2}(K_{t_{1}}||\ldots ||K_{t_{m}})||H_{2}(ID_{t_{1}}||\ldots ||ID_{t_{m}})||T),$$

where T is the current timestamp, $K_{t_{j}}$ and $ID_{t_{j}}$ indicate the user key and identity of the user set, respectively. CA randomly selects grand to generate the multicast session key $cuk=H_{1}(csk||grand)$.

### 5.3. Data Encryption

**Figure 2:**
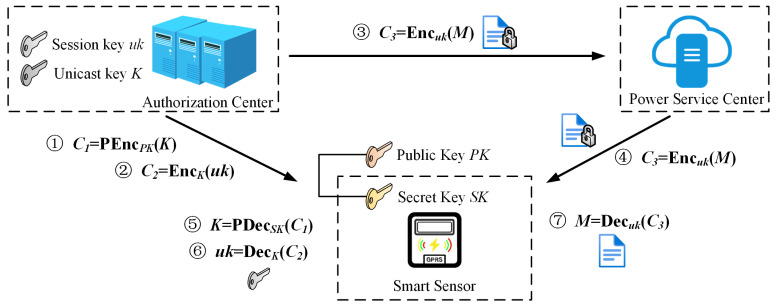
Workflow of the secure data unicast service.

**(a) Unicast encryption:** As shown in Figure 2, CA uses the user’s public key $PK_{i}^{j}$ to encrypt the user key to obtain $C_{i}^{j}=PEnc({PK}_{i}^{j},K_{i}^{j})$, and then uses the unicast key to encrypt the session key to obtain $Enc(K_{i}^{j},uk_{i}^{j})$, and finally uses the session key to encrypt the plaintext M to obtain $Enc(uk_{i}^{j},M)$, where PEnc represents identity based public key encryption algorithm, and Enc represents any symmetric encryption algorithm.

**Figure 3:**
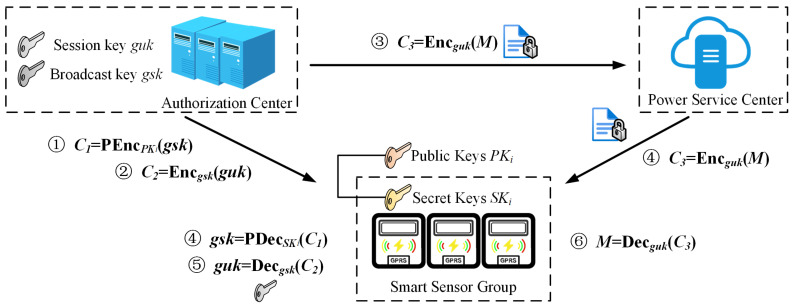
Workflow of the secure data broadcast service.

**(b) Broadcast encryption:** As shown in Figure 3, CA uses the user’s public key ${PK}_{i}^{j}$ to encrypt the broadcast key to obtain $C=PEnc({PK}_{i}^{j},gsk)$, and then uses the broadcast key to encrypt the broadcast session key to obtain Enc(gsk,guk), and finally uses the broadcast session key to encrypt the plaintext M to obtain Enc(guk,M).

**Figure 4:**
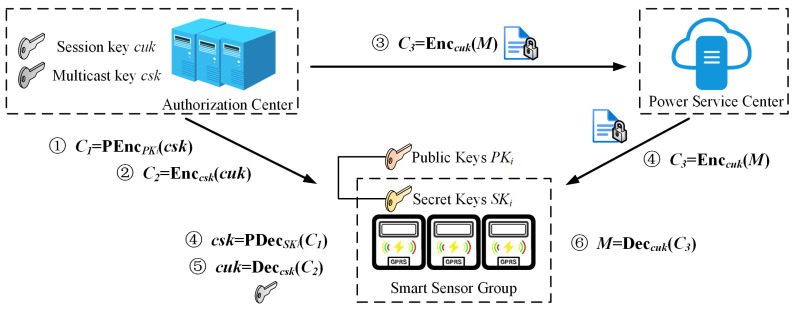
Workflow of the secure data multicast service.

**(c) Multicast encryption:** As shown in Figure 4, CA uses the user’s public key ${PK}_{i}^{j}$ to encrypt the multicast key to obtain $C^{\prime }=PEnc({PK}_{i}^{j},csk)$, and then uses the multicast key to encrypt the multicast session key to obtain Enc(csk,cuk), and finally uses the multicast session key to encrypt the plaintext M to obtain Enc(cuk,M).

### 5.4. User Authentications

**Figure 5:**
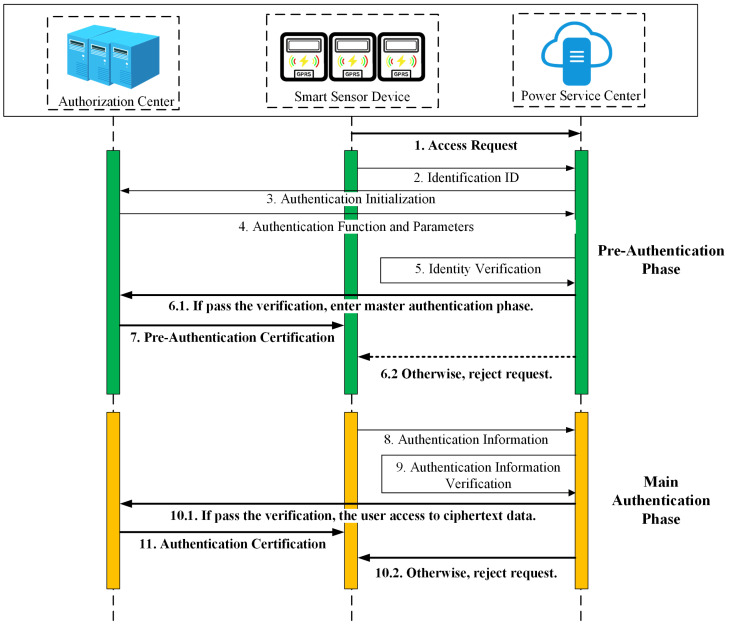
Workflow of the user authentication phase.

If the user wants to make a data access request to the power service center, it first verifies the legitimacy of the identity. As shown in Figure 5, user authentications include pre-authentication and main authentication. The specific process is as follows.

**Pre-authentication:** The power service center uses the hash function disclosed by CA in the initialization stage to hash the user identity and obtain the pre-authentication array $A^{\prime }$ generated by the Bloom filter. The power service center pre-verifies the user identity: If $A^{\prime }\subseteq A$, the user passes the authentication and obtains the pre-authentication certification. The power service center calculates $R_{B}=msk\cdot pk_{i}^{j}=x\cdot x{\left(t_{i}^{j}\right)}^{-1}\cdot P$.

**Main authentication:** After passing the pre-authentication, the user uses his private key and the system master public key to calculate the shared key $R_{A}=sk_{i}^{j}\times mpk$, and calculates

$$Auth=H_{1}(R_{A}|\left|T_{A}\right||timestamp).$$

Then, the user sends Auth||timestamp to the power service center. The power service center receives Auth||timestamp at ${timestamp}^{\prime }$ moment and firstly checks whether the time interval between timestamp and ${timestamp}^{\prime }$ meets $|timestamp^{\prime }-timestamp|\leq \Delta T$, where ΔT represents the transmission delay. If the timestamp falls within the accepted time window, the power service center calculates $Auth^{\prime }=H_{1}(R_{B}||T_{A}||timestamp)$, if $Auth^{\prime }=Auth$, then it proves that the user has the correct certification and public and private key pair, and the user successfully passes the authentication.

### 5.5. Data Decryption

**(a) Unicast decryption:** As shown in Figure 2, the smart sensor device uses its private key $SK_{i}^{j}$ to calculate ${PK}_{i}^{j}=PDec({SK}_{i}^{j},C_{i}^{j})$, decrypts the unicast key, then the session key $uk_{i}^{j}$ by using the user key, and finally decrypts the plaintext M by using the session key.

**(b) Broadcast decryption:** As shown in Figure 3, the smart sensor device uses its own private key $SK_{i}^{j}$ to calculate $gsk=PDec({SK}_{i}^{j},C)$ to obtain the user’s broadcast key, and then the broadcast session key is obtained by using the broadcast key. Finally, the plaintext M is decrypted by using the broadcast session key.

**(c) Multicast decryption:** As shown in Figure 4, the smart sensor device uses its own private key $SK_{i}^{j}$ to calculate $csk=PDec\left({SK}_{i}^{j},C^{\prime }\right)$ to obtain the user’s multicast key, and then multicast session key cuk is obtained by using the multicast key. Finally, the plaintext M is decrypted by using the multicast session key.

### 5.6. Dynamic Updating of Terminal Devices

#### 5.6.1. Smart Sensor Device Addition

**Figure 6:**
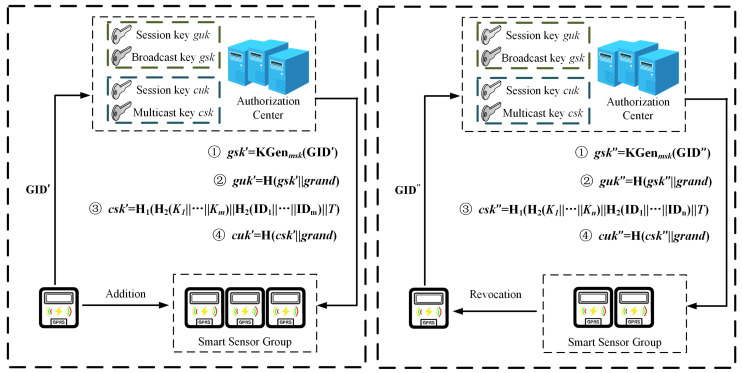
Workflow of the dynamic key management.

As shown in Figure 6, when a new user is added to the system, the system generates the public and private keys pair and user key and updates the broadcast key, broadcast session key, multicast key, and multicast session key. The public key, private key, and user key follow the steps for key generation.

**(a) Updating the broadcast key and broadcast session key:** CA generates the broadcast key $gsk^{\prime }=KGen(msk,GID)$ using the master public key mpk, master private key msk, and identity of the existing device GID. AC selects grand at random to generate the broadcast session key $guk^{\prime }=H_{1}(gsk||grand)$.

**(b) Updating the multicast key and multicast session key:** When the power service center wants to multicast with a subset of users containing new users, it firstly recalculates the hash value of the user keys and identities for users: $csk^{\prime }=H_{1}(H_{2}(K_{t_{1}}||\ldots ||K_{t_{m}})||H_{2}(ID_{t_{1}}||\ldots ||ID_{t_{m}})||T)$, where T indicates the current timestamp, and $K_{t_{j}},ID_{t_{j}}$ indicates the user keys and identities of users, respectively. CA randomly selects grand to generate the multicast session key $cuk^{\prime }=H_{1}(csk^{\prime }||grand)$.

#### 5.6.2. Smart Sensor Device Revocation

As shown in Figure 6, when a smart sensor device is revoked, its public key, private key, and authentication certification will expire, and it can no longer be used for data access.

**(a) Updating the broadcast key and broadcast session key:** When a smart device is removed, the broadcast key needs to be recalculated. According to the key generation algorithm, the broadcast key $gsk^{\prime \prime }$ is recalculated, and then the broadcast session key $guk^{\prime \prime }$ is generated.

**(b) Updating the multicast key and multicast session key:** When a smart device is removed, the multicast key needs to be recalculated. According to the key generation algorithm, the multicast key ${csk}^{\prime \prime }$ is recalculated, and then the multicast session key ${cuk}^{\prime \prime }$ is generated.

**(c) Smart sensor device migration:** When a subset of user devices is migrated from one gateway to another gateway device, CA needs to recalculate the hash values of the user keys and identities for all nodes on the path from this subset of users to the root node, and then obtain a new multicast key $csk^{\prime \prime }$, which is based on the current new timestamp $\tilde{T}$. $csk^{\prime \prime }$ is broadcast to users in this subset with the previous multicast session key, and upon receiving the message, users decrypt and update the multicast session key $cuk^{\prime \prime }$.

### 5.7. Fault Recovery

The cloud server maintains an active device list L and monitors connection intervals Δt for all client devices. For any device $d_{i}$ with Δt > τ, where τ denotes the maximum allowable inactive duration, the cloud server automatically revokes its authentication credentials and removes $d_{i}$ from L.

Similarly, the CA implements regular check with period τ. At each interval, CA sends status requests to cloud server, analyzes status feedback, and repairs or replaces broken-down cloud devices. A failure log matrix $M_{fail}$ is held by CA for post-incident analysis.

## 6. Security Analysis

This section formally analyzes the security properties of the proposed protocol. The security relies on the hardness of the elliptic curve discrete logarithm problem (ECDLP), the computational Diffie–Hellman (CDH) problem on elliptic curves, and the security of the underlying identity-based encryption (IBE) scheme (PEnc,PDec) and symmetric encryption scheme (Enc,Dec). The hash functions $H,H_{1},H_{2}$ are assumed to be cryptographically secure (e.g., behaving as random oracles).

### 6.1. Forward Security

**Theorem 1.**  *Forward security ensures that a smart sensor device $SM_{r}$ (with identity $ID_{r}$), once revoked from a group at time $T_{rev}$, cannot decrypt messages intended for the group after $T_{rev}$. This means $SM_{r}$ cannot obtain any newly generated group keys (e.g., gsk′, csk′) or session keys (e.g., guk′, cuk′).*

**Proof.**  Consider the broadcast key gsk. When $SM_{r}$ is revoked, a new broadcast key gsk′ is generated by CA using $gsk\prime =KGen\left(msk,GID\prime \right)$ where GID′ represents the identities of the remaining authorized devices. This gsk′ is then used to derive a new broadcast session key $guk\prime =H_{1}\left(gsk^{\prime }||grand\prime \right)$ The plaintext M is encrypted as $Enc\left(guk\prime ,M\right)$ The key gsk′ is distributed to authorized users by encrypting it with their respective public keys $PK_{u}$ (derived from $ID_{u}$) using the IBE scheme: $C_{u}=PEnc\left(PK_{u},gsk\prime \right)$.

Since $SM_{r}$ is revoked, its identity $ID_{r}$ is not part of GID′, and CA will not provide $SM_{r}$ with $PEnc\left(PK_{r},gsk\prime \right)$ Even if $SM_{r}$ possesses its old private key $sk_{r}$ it cannot decrypt $C_{u}$ for any $ID_{u}\in GID\prime$ (assuming $ID_{u}\neq ID_{r}$) to obtain gsk′, due to the security of the IBE scheme. Without gsk′, $SM_{r}$ cannot compute guk′ via $H_{1}\left(gsk^{\prime }||grand\prime \right)$ (as grand′ is fresh and $H_{1}$ is one-way). Therefore, $SM_{r}$ cannot decrypt $Enc\left(guk\prime ,M\right)$.

The argument for the multicast key csk′ is similar. When $SM_{r}$ is revoked from a multicast group, a new csk″ is computed based on the remaining members’ keys and identities, and a new timestamp. This csk″ is distributed encrypted with the previous multicast session key cuk Since $SM_{r}$ is no longer part of the group, it will not receive this update, or if it does, the subsequent session key cuk″ will be derived from csk″ which it cannot obtain if csk″ is re-encrypted using IBE for the new group. If csk″ is broadcast using the old cuk, then the subsequent cuk″ derived from csk″ and a new grand″ will be unknown to $SM_{r}$. The core idea is that new keys are generated that $SM_{r}$ does not have the components to derive or decrypt.

Thus, the protocol ensures forward security provided the IBE scheme is secure and hash functions are one-way. An adversary controlling $SM_{r}$ cannot gain access to future group communications. □

### 6.2. Backward Security

**Theorem 2.**  *Backward security ensures that a newly added smart sensor device $SM_{n}$ (with identity $ID_{n}$) at time $T_{add}$ cannot decrypt messages encrypted for the group before its addition.*

**Proof.**  Before $SM_{n}$ joins, group communications use keys like $gsk_{old}$ (and $guk_{old}$) or $csk_{old}$ (and $cuk_{old}$). These keys were generated based on the identities and keys of members existing before $T_{add}$. For instance, $gsk_{old}=KGen\left(msk,GID_{old}\right)$, where $ID_{n}\notin GID_{old}$.

When $SM_{n}$ joins, it receives its own private key $sk_{n}$, user key $K_{n}$, and session key $uk_{n}$. However, $SM_{n}$ does not receive past group keys like $gsk_{old}$ or $csk_{old}$. The IBE-encrypted $gsk_{old}$ was distributed only to members of $GID_{old}$. Since $ID_{n}$ was not in $GID_{old}$, $sk_{n}$ cannot be used to decrypt ciphertexts containing $gsk_{old}$.

The user keys $K_{u}=H\left(msk||ID_{u}||R_{u}||T\right)$ are unique to each user and timestamp. A new user $SM_{n}$ cannot derive past user keys of other members due to the one-way nature of H and the secrecy of msk and other users’$R_{u}$. Similarly, past multicast keys $csk_{old}=H_{1}\left(H_{2}\left(K_{t_{1}}||\ldots \right)||H_{2}\left(ID_{t_{1}}||\ldots \right)||T_{old}\right)$ depend on keys and identities of the old group and an old timestamp, which $SM_{n}$ cannot reconstruct.

Therefore, $SM_{n}$ cannot access messages encrypted prior to it joining the group, ensuring backward security. This relies on the IBE security and the one-way property of hash functions. □

### 6.3. Replay Attack Resistance

**Theorem 3.**  *The protocol resists replay attacks during user authentication. The user sends $Auth=H_{1}\left(R_{A}|\left|T_{A}\right||timestamp\right)$ and timestamp to the power service center. $R_{A}=sk_{i}^{j}\cdot mpk$.*

**Proof.**  The power service center (server) receives $\left(Auth,timestamp\right)$ at its current time timestamp′ It first verifies the freshness of the timestamp by checking if $\left|timestamp\prime -timestamp\right|\leq \Delta T$, where ΔT is a predefined small interval for network delay.

If an adversary intercepts a valid $\left(Auth_{1},timestamp_{1}\right)$ and replays it at a significantly later time $timestamp\prime_{2}$, then $\left|timestamp\prime_{2}-timestamp_{1}\right|>\Delta T$ The server will detect this stale timestamp and reject the message.

If the adversary attempts to use a fresh $timestamp_{2}$ with the old $Auth_{1}$, the server will compute $Auth\prime_{check}=H_{1}\left(R_{B}|\left|T_{A}\right||timestamp_{2}\right)$ (where $R_{B}=msk\cdot pk_{i}^{j}=R_{A}$). Since $timestamp_{2}\neq timestamp_{1}$ and $H_{1}$ is collision-resistant, $Auth_{1}=H_{1}\left(R_{A}|\left|T_{A}\right||timestamp_{1}\right)$ will not be equal to $Auth\prime_{check}$ (except with negligible probability). Thus, the authentication will fail.

To successfully replay with a fresh timestamp $timestamp_{2}$, the adversary would need to compute a new $Auth_{2}=H_{1}\left(R_{A}|\left|T_{A}\right||timestamp_{2}\right)$. This requires knowledge of $R_{A}=sk_{i}^{j}\cdot mpk$. Since the user’s private key $sk_{i}^{j}$ is secret, and computing $sk_{i}^{j}\cdot mpk$ without $sk_{i}^{j}$ is hard (related to the CDH problem, given $pk_{i}^{j}=sk_{i}^{j}P$ and mpk=xP), the adversary cannot forge a valid $Auth_{2}$.

The inclusion of a fresh timestamp in the hash computation for Auth and the server’s freshness check effectively prevent replay attacks. □

### 6.4. Impersonation Attack Resistance

**Theorem 4.**  *An adversary attempts to impersonate a legitimate smart sensor device $SM_{u}$ (with identity $ID_{u}$ and private key $sk_{u}$) to the power service center.*

**Proof.**  To impersonate $SM_{u}$, the adversary must successfully complete the authentication process. This involves computing $Auth=H_{1}\left(R_{A}|\left|T_{A}\right||timestamp\right)$, where $R_{A}=sk_{u}\cdot mpk$. The adversary knows $ID_{u}$, mpk=xP, and $pk_{u}=sk_{u}P$. The system master key x and user private key $sk_{u}$ are secret.

The private key $sk_{u}$ is computed as $x\cdot {\left(t_{u}\right)}^{-1}$, where $t_{u}=H_{1}\left(ID_{u}||hid,N\right)+x$. Thus, $R_{A}=\left(x\cdot {\left(t_{u}\right)}^{-1}\right)\cdot \left(xP\right)=x^{2}{\left(t_{u}\right)}^{-1}P$.

An adversary faces the following difficulties:

Deriving $sk_{u}$ from $pk_{u}=sk_{u}P$: This is the ECDLP, which is assumed to be hard.

Deriving x from mpk=xP: This is also the ECDLP.

Computing $R_{A}=sk_{u}\cdot mpk$ directly from $pk_{u}$ and mpk without knowing $sk_{u}$ or x: This is computationally equivalent to solving the CDH problem (given $P,sk_{u}P,xP$, compute $sk_{u}xP$). While $R_{A}$ is not exactly $sk_{u}xP$, computing $x^{2}{\left(H_{1}\left(ID_{u}||hid,N\right)+x\right)}^{-1}P$ without x is infeasible.

The pre-authentication step using the Bloom filter adds another layer. If the adversary’s chosen (or forged) identity $ID_{adv}$ is not in the authorized set encoded in the Bloom filter A on the server, $A\prime_{adv}⊈A$, and pre-authentication fails. Even if a Bloom filter collision occurs for a random $ID_{adv}$ (with small probability ϵ), the subsequent main authentication requiring $R_{A}$ will fail.

Since the adversary cannot compute $sk_{u}$ or $R_{A}$ without breaking underlying hard problems (ECDLP or CDH), it cannot generate a valid Auth message. Therefore, the protocol is resistant to impersonation attacks. □

## 7. Performance Analysis

### 7.1. Theoretical Analysis

This section will be analyzed in terms of computational cost and communication cost. In computational cost analysis, we focus only on the bilinear pairing, multiplication, hashing, and modular inverse algorithms performed by every device, and ignore other lightweight operations. We represent P as a bilinear pairing operation, m as a multiplication operation, h as a hash operation, and r as a modular power operation. In the registration phase, the power service center generates the unicast key for every smart grid device, which requires three multiplications, three hashes, and one modular power, namely 3m+3h+1r. The power service center needs 2h operations to generate the broadcast key, and 2h operations to generate the multicast key. In the encryption phase, unicast encryption, broadcast encryption, and multicast encryption all require one asymmetric encryption operation and two symmetric encryption operations. In the authentication phase, CA authenticates user identity by one multiplication and one hash. In communication cost analysis, CA generates the private key, session key, broadcast key, broadcast session key, multicast key, and multicast session key for the user. Their key’s bit length is λ. During data access, the user downloads the ciphertext from the power service center. We will ignore other transmitted data.

Table 1 shows the cost comparison of the certification process between the proposed protocol and the protocols of MAH [26], WAN [28], and ZHA [14]. In our protocol, CA requires one multiplication and one hash operation to verify the validity of the terminal device. Therefore, the time consumed by CA authentication increases linearly with the increasing number of terminal devices. As shown in Table 2, the cost of the proposed protocol is lower than other protocols, both in terms of computation cost and communication cost. MAH protocol has the highest computational cost, and WAN protocol has the highest communication cost.

Table 2 shows the cost comparison of encryption and decryption between the proposed protocol and the protocols of CHE [22], ACH [23], and KUM [27]. The protocol of CHE and KUM has low encryption costs but requires a lot of bilinear pairing operations in the key generation and decryption stage, which is not suitable for resource-limited terminal devices in smart grids. In the decryption cost, the proposed protocol has a lower cost.

### 7.2. Experimental Analysis

In this section, we conduct some experiments to prove that the protocol we designed is efficient. We compare the proposed scheme with the schemes in CHE, ACH, and KUM in terms of encryption cost and decryption cost.

In our experiments, we used a computer with an Intel (R) Core (TM) i9-10920X CPU @ 3.50 GHz to simulate the CA in the design system. The cloud server was deployed on a 1-core, 2 GB RAM virtual machine with a high-I/O 40 GB system disk. We employ real-world smart grid terminal devices to evaluate the proposed protocol. Specifically, converged terminals use SCM701 MCUs with 4 GB ROM and 1 GB RAM, load control terminals leverage AT91SAM9G25-CU MCUs with 256 MB ROM and 64 MB RAM, and multi-function terminals integrate i.MX6 Dual-Core MCUs with 4 GB ROM and 512 MB RAM. In the experiment, the C++ programming language is used to implement the designed protocol, and the PBC library is used to multiply and modular exponents of elliptic curves. The hash function in our experiment is SHA-256. All tests were conducted under Ubuntu 20.0, while network conditions were emulated using Linux Traffic Control to impose 100 Mbps bandwidth and 50 ms latency constraints. These configurations are identical to smart grid environments, thereby demonstrating the scheme’s effectiveness in real-world scenarios.

**Figure 7:**
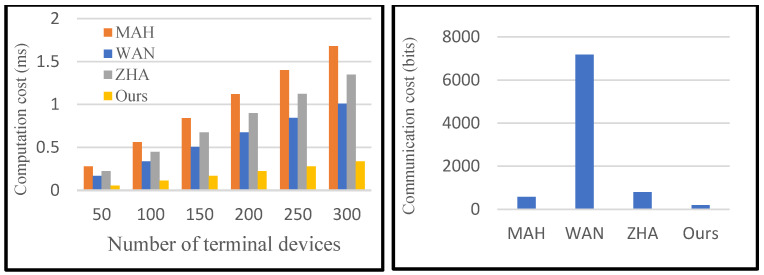
Comparison of computation cost and communication cost of different protocols.

As shown in Figure 7, both the computation and communication costs of the proposed protocol are less than other authentication protocols. Since the Bloom filter is introduced to encode the identities of smart sensor devices, the proposed protocol reduces the computation and communication cost of the certification phase. Detailed experimental results are shown in Table 3 and Table 4. With the increasing number of terminal devices, the advantage of our protocol becomes more obvious. Specifically, the proposed protocol is 73.94% superior to others on average in terms of computation and 79.98% in terms of communication.

**Figure 8:**
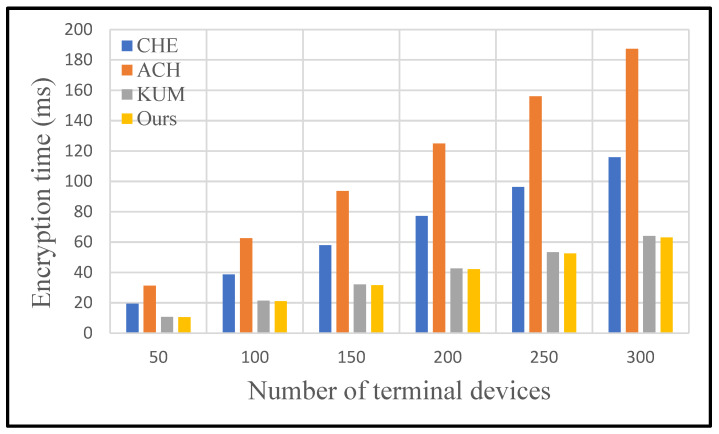
Comparison of encryption time of different protocols.

**Figure 9:**
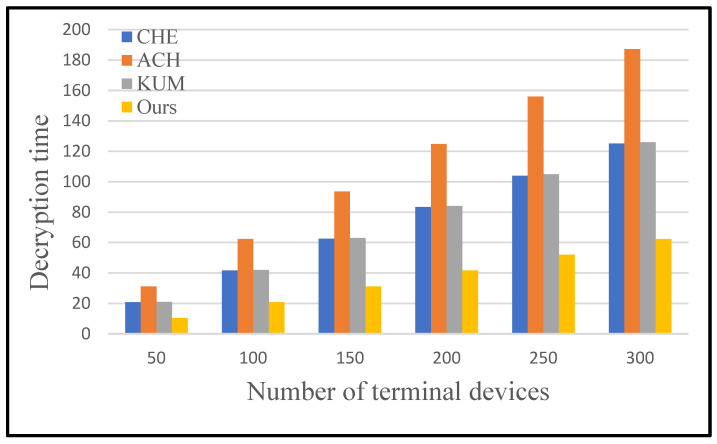
Comparison of the decryption time of different protocols.

Figure 8 and Figure 9 show comparisons of data encryption and decryption costs for each scheme with the increasing number of terminal devices. The proposed scheme is obviously superior to other schemes in terms of encryption and decryption cost. Detailed experimental results are shown in Table 5 and Table 6. Specifically, the running time of unicast encryption and multicast encryption increases linearly with the increase in terminal devices. Broadcast encryption does not consume excessive uptime due to changes in the number of devices. No matter how many devices are added, broadcast encryption is calculated only once using the device’s identity.

For the encryption process, the proposed protocol performs best among the four secure data communication protocols. KUM protocol also performs well with the increasing number of smart sensor devices, followed by CHE protocol, while ACH protocol showcases the worst performance. Specifically, the proposed protocol is 37.77% superior to others on average in terms of encryption cost.

For the decryption process, the proposed protocol is the most efficient protocol among the four secure data communication protocols as well. Particularly, the KUM protocol and CHE protocol demonstrate similar performance on efficiency in the decryption phase, which significantly falls behind our protocol. In terms of decryption efficiency, the ACH protocol is the worst protocol as well. Specifically, the proposed protocol is 55.75% superior to others on average in terms of decryption cost.

According to the evaluations above, our protocol showcases comprehensive advantages in the secure data communication process. The proposed protocol obviously demonstrates the best performance on authentication, encryption, and decryption progress. In conclusion, the proposed protocol provides an effective and efficient solution for secure data communication in smart grid scenarios.

## 8. Conclusions

In this paper, we propose a novel secure data communication protocol for sensor groups in the smart grid system. The proposed protocol leverages the symmetric encryption method to transmit obscured data and utilizes the identity-based encryption method to share group secret keys. To improve communication efficiency, the identities of authorized users are encoded by the Bloom filter, and a cloud-aided pre-verification procedure is introduced. Correspondingly, a novel dynamic update mechanism of the group secret key is designed in smart grid scenarios. When the sensor group is changed, the proposed mechanism utilizes lightweight operations to implement dynamic updates of the group secret key. Theoretical analysis demonstrates that our protocol achieves forward and backward security of a dynamic sensor group and has the capability to resist the replay attack and impersonation attack. Experimental evaluation indicates that our protocol performs better than the state-of-the-art protocols. Specifically, for computation cost, the proposed protocol is 73.94% superior to others on average in the authentication process, 37.77% in the encryption process, and 55.75% in the decryption process. For communication cost, the proposed protocol is 79.98% superior to others on average in the authentication process.

## Figures and Tables

**Table 1:** Theoretical cost comparison of the certification process for different schemes.

Protocol	Computation Cost	Communication Cost
MAH	5m+5h	3λ+96
WAN	6m+3h	$3\lambda^{2}+2\lambda +64$
ZHA	5m+4h	5λ
Ours	m+h	λ+32

**Table 2:** Theoretical cost comparison of encryption and decryption for different schemes.

Protocol	Computation Cost	Communication Cost
CHE	5r	2P+nm
ACH	3P+4m	3P
KUM	m+3r	2P+r
Ours	P+2m+h+r	P

**Table 3:** Computation cost comparison of the certification process for different schemes.

Protocol	Number of Devices					
	50	100	150	200	250	300
MAH	0.28 ms	0.56 ms	0.84 ms	1.12 ms	1.40 ms	1.68 ms
WAN	0.16 ms	0.34 ms	0.51 ms	0.67 ms	0.84 ms	1.01 ms
ZHA	0.22 ms	0.45 ms	0.67 ms	0.90 ms	1.12 ms	1.35 ms
Ours	0.06 ms	0.11 ms	0.17 ms	0.22 ms	0.28 ms	0.34 ms

**Table 4:** Communication cost comparison of the certification process for different schemes.

Protocol	MAH	WAN	ZHA	Ours
**Cost**	576 bits	7184 bits	800 bits	192 bits

**Table 5:** Computation cost comparison of the encryption process for different schemes.

Protocol	Number of Devices					
	50	100	150	200	250	300
CHE	19.29 ms	38.58 ms	57.87 ms	77.16 ms	96.25 ms	115.74 ms
ACH	31.20 ms	62.44 ms	93.61 ms	124.87 ms	156.34 ms	187.24 ms
KUM	10.65 ms	21.30 ms	31.95 ms	42.60 ms	53.25 ms	63.90 ms
Ours	10.50 ms	21.00 ms	31.50 ms	42.00 ms	52.51 ms	63.12 ms

**Table 6:** Computation cost comparison of the decryption process for different schemes.

Protocol	Number of Devices					
	50	100	150	200	250	300
CHE	20.85 ms	41.57 ms	62.55 ms	83.41 ms	104.02 ms	125.13 ms
ACH	31.20 ms	63.91 ms	94.34 ms	123.99 ms	154.97 ms	188.01 ms
KUM	21.05 ms	42.17 ms	63.18 ms	84.06 ms	105.21 ms	126.20 ms
Ours	10.39 ms	20.82 ms	31.27 ms	41.60 ms	51.98 ms	62.42 ms

## Data Availability

The raw data supporting the conclusions of this article will be made available by the authors on request.
